# What have genome-wide association studies taught us about cGMP and blood pressure regulation?

**DOI:** 10.1186/2050-6511-14-S1-O5

**Published:** 2013-08-29

**Authors:** Christopher Newton-Cheh

**Affiliations:** 1 Massachusetts General Hospital, Boston, MA, USA; 2 Broad Institute of Harvard and MIT, Cambridge, MA, USA

## Background

Hypertension (HTN) is a major worldwide cause of stroke, heart failure, myocardial infarction, and chronic kidney disease. Mechanisms of blood pressure regulation have previously been elucidated from the study of animal models, Mendelian hyper- and hypo-tension in humans, and drug treatment trials. More recently, genome-wide association studies (GWASs) have offered unbiased approaches to identify novel mechanisms contributing to blood pressure control. We and others have reported candidate gene and GWASs that have identified common genetic variants of modest effect on blood pressure that fall in pathways involving natriuretic peptide and nitric oxide signalling [1-3].

## Results

Common variant rs5068 in the 3’ UTR of *NPPA*, which encodes the atrial natriuretic peptide (ANP), is associated with higher plasma ANP and lower blood pressure and odds of hypertension [2]. We have now completed a genotype-directed physiologic study of 8 heterozygotes and 23 major homozygotes demonstrating that the variant alters the “set point” of ANP (p=0.02) but does not alter the ANP response to high vs low sodium diet (p<0.0001) or to intravenous saline infusion (p<0.0001, in press); additional evidence for the mechanism by which this variant acts will be presented in a separate abstract. Downstream of the natriuretic peptide system, a common noncoding variant at a locus including *NPR3*, encoding the natriuretic peptide clearance receptor, has been associated with blood pressure although it is not associated with plasma ANP or BNP [3]. A common variant at the locus including *ENOS*, encoding endothelial nitric oxide synthase, is associated with blood pressure and hypertension [4,5]. Lastly, an intronic variant in *GUCY1A3*, encoding the alpha subunit of soluble guanylate cyclase, is associated with blood pressure and myocardial infarction risk.

## Conclusion

The natriuretic peptide and nitric oxide systems are important contributors to blood pressure regulation in humans and represent strong targets for pharmacologic intervention to reduce the burden of disease from hypertension.
